# Exploring Reactions to Disrespect and Sensitivity to Social Rejection in Older Adults

**DOI:** 10.1093/geroni/igaa057.1481

**Published:** 2020-12-16

**Authors:** Amanda Chappell, Kelly Smith, Jenessa Steele, JoNell Strough

**Affiliations:** 1 West Virginia University, Morgantown, West Virginia, United States; 2 Radford University, Radford, Virginia, United States

## Abstract

Disrespect involves having low regard or low esteem for someone. Disrespect is a universal experience and has the potential to negatively impact relationships and fosters anger and aggression (Hawkins, 2015; Shwalb & Shwalb, 2006). In the current study, younger (ages 19-25) and older adults (ages 50-77) imagined a person they knew had disrespected them in six different hypothetical situations. For each situation, participants rated their emotional reactivity. Participants also indicated their sensitivity to social rejection (i.e. being left out/excluded). The primary research questions for this study included: do reactions to disrespect differ based on age? Also, does one’s relationship status with a disrespect perpetrator matter? In the current study, participants had a stronger emotional reaction to disrespect when imagining the disrespect perpetrator was someone close to them rather than someone not close to them, regardless of age. An age by gender by relationship closeness ANOVA revealed three significant main effects: of age (older are less sensitive), gender (males are less sensitive), and relationship closeness (those more distant to perpetrator are less sensitive) on sensitivity to social rejection. Sensitivity to social rejection mediates the relationship between closeness to a disrespect perpetrator and emotional reaction, even after controlling for age as a covariate, p<.001. The findings of this study highlight the component of rejection that is typically involved within disrespect situations and may help to explain just why disrespect feels so hurtful. These findings also highlight that there may be some protection from disrespect based on age or gender.

